# Enhanced Sensitive Love Wave Surface Acoustic Wave Sensor Designed for Immunoassay Formats

**DOI:** 10.3390/s150510511

**Published:** 2015-05-05

**Authors:** Mihaela Puiu, Ana-Maria Gurban, Lucian Rotariu, Simona Brajnicov, Cristian Viespe, Camelia Bala

**Affiliations:** 1 LaborQ, University of Bucharest, 4-12 Regina Elisabeta Blvd., 030018 Bucharest, Romania; E-Mails: elenamihaela.puiu@g.unibuc.ro (M.P.); amgurban@yahoo.com (A.-M.G.); lucian.rotariu@g.unibuc.ro (L.R.); 2 Department of Analytical Chemistry, University of Bucharest, 4-12 Regina Elisabeta Blvd., 030018 Bucharest, Romania; 3 National Institute for Laser, Plasma and Radiation Physics, 409 Atomistilor Blvd., RO-077125 Magurele, Romania; E-Mails: simonabrajnicov@yahoo.com (S.B.); cristian.viespe@inflpr.ro (C.V.)

**Keywords:** surface acoustic wave, surface plasmon resonance, aflatoxin B1, immunoassay

## Abstract

We report a Love wave surface acoustic wave (LW-SAW) immunosensor designed for the detection of high molecular weight targets in liquid samples, amenable also for low molecular targets in surface competition assays. We implemented a label-free interaction protocol similar to other surface plasmon resonance bioassays having the advantage of requiring reduced time analysis. The fabricated LW-SAW sensor supports the detection of the target in the nanomolar range, and can be ultimately incorporated in portable devices, suitable for point-of-care testing (POCT) applications.

## 1. Introduction

Love wave surface acoustic wave (LW-SAW) sensors are the most recent developed devices designed for integration in “lab-on-a-chip” systems, being extended in the last years to DNA, antibodies and protein analysis in solution. A surface acoustic wave represents a mechanical acoustic wave that propagates to a confined area of a cut piezoelectric crystal [1]. Love waves propagate near the surface of the piezoelectric material which supports shear horizontal waves, if it is deposited on the top of the piezoelectric substrate and over the layer with a lower shear velocity. The velocity and the amplitude of the wave are strongly dependent on the changes occurring in the media near the surface [1]. During a LW-SAW assay, the polarized transversal waves (resulted from the electrical signal converted at the interdigital transducers, IDTs), are propagating along the sensing surface due to the piezoelectric properties of the substrate [2,3]. The changes induced near the sensor’s surface by specific biological interactions occurring when a bioreceptor is immobilized onto this surface could be exploited in order to increase the sensitivity of the SAW approach [4]. Since the wave’s travel is restricted to an independent guiding layer, the acoustic energy is concentrated within the guiding layer and not in the bulk of the piezoelectric crystal. Therefore, the sensitivity of the sensor is mainly influenced by the guiding layer’s characteristics and design [5]. It may appear that for *in situ* measurements in solution, label free detection and real-time monitoring, LW-SAW sensors are not competitive with the surface plasmon resonance (SPR) technology, due to the available commercial SPR sensors and their easy-to-build Kretschmann configuration [1,6]. The detection principle of the SPR sensors lies in the changes in the refractive index due to the mass changes near the surface [7]. In this respect, the main advantage of the SAW sensors over the SPR sensors consists in their enhanced sensitivity to changes in density, mass and viscosity [5]. They can be amenable in miniaturized, cost-effective and portable devices needed in point-of-care testing (POCT), enough to be used at the primary care level with no laboratory infrastructure.

Aflatoxin B1 (AFB1) is one of the most toxic natural products from the mycotoxins class, contaminating human food and animal feed [8]. The mechanism of the AFB1 induced toxicity with respect to human and animal health is explained by the fact that in the liver, AFB1, in its highly reactive form, aflatoxin B1-8-9-epoxide, binds exclusively to guanyl residues from DNA resulting in the aflatoxin B1-N7-guanine adduct (AFB1-N7-Gua). This DNA adduct is considered the main factor responsible for the initiation of carcinogenesis. Along with the DNA adducts, AFB1 binds other plasma proteins to form, for example, AFB1-albumin adduct whose level is correlated with the amount of AFB1-N7-Gua [9]. For this reason, the measurement of AFB1-albumin level or of another AFB1-protein bioconjugate may be used as a biomarker of cumulative AFB1 exposure [10]. Enzyme-linked immunosorbent assay (ELISA) is a wide spread, highly selective technique based on the biorecognition of antibody-antigen (Ab-Ag) coupling [11], which is currently used for the detection and quantification of aflatoxins in food, environmental and blood samples. The main disadvantages of the ELISA assays that must be overcome are the time-consuming steps and the need for laboratory skilled personnel. In this respect, novel methods that can allow detecting biomarkers for AFB1 exposure in blood samples using miniaturized and portable devices such as SAW sensors are feasible and urgently required.

AFB1 itself, being a low molecular weight compound (LMW), below 300 Da, is difficult to be detected directly in solution with the conventional SPR sensor or a SAW sensor. The reason is that both type of sensors, whose detection principles lie mainly in responses to mass variation, provide poor signals for LMW targets when working in solution. In blood or animal food samples, AFB1 is present as a protein–bound conjugate, with a high molecular weight (HMW), and therefore it becomes suitable to develop a detection protocol for that target.

We report in this paper an ELISA-like method for direct detection of AFB1-BSA bioconjugate by immobilizing the corresponding antibody to a LW-SAW sensor surface. The conjugate AFB1-BSA is a HMW target, which can be directly monitored with an SPR sensor. The immuno-interaction protocol was checked firstly with a SPR sensor and the Ab-Ag binding was monitored with both LW-SAW and SPR sensors. The analytical performances of the LW-SAW sensor were optimized for a simple and easy to implement binding assay, and compared with those of a conventional SPR sensor, in similar conditions. Our fabricated LW-SAW sensor was easy-to-use and less time consuming than the SPR sensor for the same Ab-Ag assay.

## 2. Experimental Section

### 2.1. Materials

11-mercaptoundecanoic acid (11-MUA) and 1-ethyl-3-(3-dimethylaminopropyl carbodiimide) hydrochloride (EDC), bovine serum albumin (BSA), hydrochloric acid and AFB1-BSA bioconjugate were purchased from Sigma–Aldrich. *N*-hydroxysuccinimide (NHS), ethanolamine and absolute ethanol were purchased from Fluka. Aflatoxin B1 (AFB1) (2a) mouse monoclonal antibody (IgG) (M.W. = 150,000) was obtained from ABCAM, Cambridge, UK as 1 mg/mL IgG solution in phosphate buffer saline (PBS) at pH 7.4 with 0.09% sodium azide. Other chemicals used were of analytical reagent grade. The optimum coupling buffer for both LW-SAW and SPR assays was 0.01 M acetate buffer solution (pH = 4.5) as a result of the pre-concentration experiments performed at several pHs, using SPR measurements. 0.1 M phosphate buffer (PBS), pH 7.5, with 0.9% (w/v) NaCl and 0.05% (v/v) Tween 20 was used as association/dissociation buffer during the interaction and washing steps. 0.1 M HCl was used in the regeneration step. All aqueous solutions were prepared with ultrapure water, obtained with a Millipore Direct Q3 system (18.2 MΩ·cm).

### 2.2. Apparatus

The SPR measurements for antibody immobilization and antibody/antigen interaction were carried out with the double channel AUTOLAB ESPRIT equipment operating at a constant temperature of 25 °C. The Autolab SPR chip with a 50-nm thick gold layer and a 5-nm titanium sublayer as the adhesive layer on glass was attached to the prism using an index-matching oil (n_d_^25^^°^^C^ = 1.518). The SPR angle shifts (Δθ_SPR_) for both channels (reference and sample) were measured in non-flow conditions, in 6 mm^2^ surface cells. The change of the SPR signal was proportional with the amount of immobilized compound, every 122 m° angle shift corresponding to 1 ng·mm^−2^ of immobilized compound. The binding curves were acquired and processed with the AUTOLAB Kinetic Evaluation 4.2.2 software.

**Figure 1:**
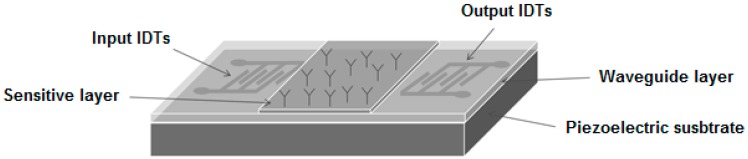
Schematic representation of the Love wave surface acoustic wave (LW-SAW) configured sensor.

**Figure 2:**
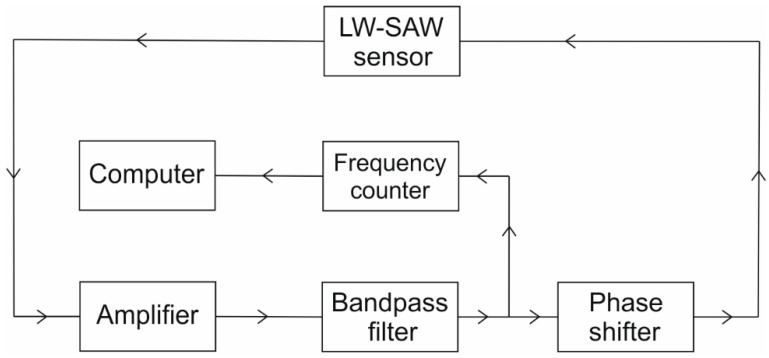
Experimental setup used to characterize the LW-SAW resonator.

Also, the measurements for antibody–antigen interaction and antibody immobilization were carried out with a LW-SAW device consisting in a piezoelectric substrate, input and output interdigital transducers, a waveguide layer, and a sensitive layer (Figure 1), together with the oscillating system (Figure 2).

AFM measurements were achieved with the XE-100 equipment from Park System, in non-contact mode, using a silicon nitride cantilever. A force of approximately 10^−12^ N was applied on the surface of the sample. 

The electrical signal connected to one of the transducers generates a surface acoustic wave which is guided through the wave-guiding layer towards the other transducer, the mechanical wave being converted in an electrical signal. 

### 2.3. Configuration of the LW-SAW Sensor

A quartz substrate was used for the fabrication of the LW-SAW sensors, due to its excellent temperature contrast compared with other piezoelectric substrates such as LiNbO_3_ and LiTaO_3_ [3,12,13]. The LW-SAW devices were fabricated on a 0.5 mm thick piezoelectric quartz crystal (Roditi International Corporation Ltd.; London, UK), Y-cut (42.75°), with propagation 90° with respect to the x-axis. The piezoelectric substrate was cut in parallelogram geometry to reduce the effect of spurious wave reflections from the edges. 

The LW-SAW consisted of two-port resonators with 50 electrode pairs with an aperture of 2500 µm and a periodicity of 45 µm. The transmitted and received IDTs were placed at a 10 mm distance from each other. The IDTs were made from 150-nm-thick gold on 10-nm-thick chromium coatings (to ensure the adhesion of the gold on quartz) by standard photolithographic methods, according to the designed configuration.

#### 2.3.1. Waveguide Mode and Deposition of the Sensitive Layers

Since LW-SAW waves often propagate slightly deeper into the substrate than Rayleigh waves, they have a lower sensitivity. If an over layer having a lower shear wave velocity is deposited on the top of the LW-SAW sensor, the energy of the shear waves is focused in the coating, and the sensitivity to surface perturbations is significantly increased. The guiding layer has the additional role of shielding the IDTs, in order to prevent the corrosion of the metal structures.

Before depositing a polymer layer on the quartz substrate, the piezoelectric surface was cleaned with methanol in an ultrasonic bath for 10 min and dried with nitrogen to remove the contaminants and to obtain a reproducible chemical surface.

A layer of polymethylmethacrylate (PMMA) (Micro Resist Technology GmbH) was applied over the clean surface by spin coating. The PMMA solution was deposited according the following program: 100 rps—5 s, 500 rps—5 s, 1500 rps—20 s, 2500 rps—40 s, 1500 rps—20 s, 500 rps—20 s, in order to ensure a uniform deposition. This was followed by a thermal solidification of the polymer at 195 °C, for 2 h. PMMA was selected as a waveguide material, due to its low shear wave acoustic velocity (1105 m/s), relativity low density (1.17 g/cm^3^), high stiffness module (1.7 GPa), and good elastic properties [14]. PMMA also shows biocompatibility, a low moisture uptake and minimal swelling in a solution [14,15]. A PMMA thickness of 2 µm was measured with a Surfcom 130A profilometer.

In order to immobilize the target biomaterials, a thin gold layer (~30 nm) was deposited onto PMMA waveguide layer, coated with gold film with an area of 9 × 10 mm between the IDTs. The gold layer was deposited by sputtering with an Agar Auto Sputter Coater 108A at a chamber pressure of 0.4 mbar and a deposition rate of 20 nm/min. The sputtering process is automatically terminated when the set thickness has been reached. The sputter coater provided a resolution of the coating thickness superior to 0.1 nm.

#### 2.3.2. Oscillating Circuit of the LW-SAW Device

In Figure 2, the oscillating system of the LW-SAW device that includes an amplifier (DHPVA-100 FEMTO; 10–60 dB, 100 MHz), a band-pass filter (Anatech Electronics B9336) and a phase shifter (IF ENGINEERING PSV-70-360-S) is presented. The frequency shift of the system was measured by the CNT-91 Pendulum counter analyzer, with Time View 2.1 software, with a 12 digits/s high resolution. The gain in impedance and phase, as a function of frequency, was measured using a network/spectrum/impedance analyzer (Agilent 4396B) with a transmission/reflection kit (Agilent 87512 A/B).

The bandpass filter was placed immediately after the amplifier in the oscillating circuit for removing any amplifier-generated harmonics that might “confuse” the frequency counter [15]_._ All the electronic components have an impedance of 50 Ω. The impedance of the LW-SAW was matched to the external circuit of 50 Ω by adding appropriate inductances.

#### 2.3.3. Design of the LW-SAW Sensor Testing Cell

**Figure 3:**
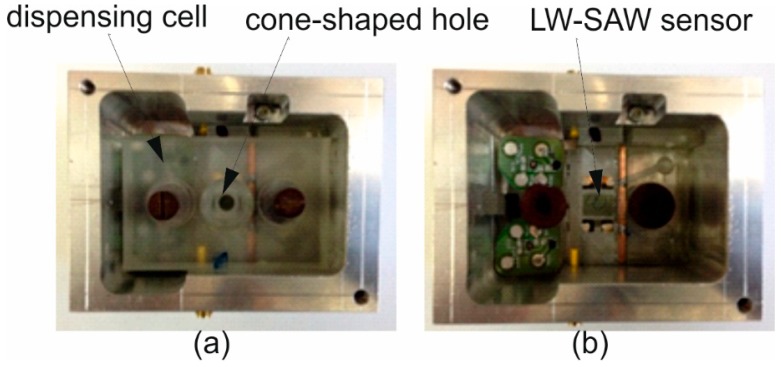
Images of the custom-made LW-SAW cell: (**a**) complete testing cell; (**b**) the testing cell without the dispensing part.

We fabricated a custom-made testing cell, which allowed the connection of the devices with the characterization equipment and suitable to perform SPR-like immunoassays. The cell was made of aluminium, and electrically grounded, in order to protect the sensor from external electromagnetic effects. In order to define a limited zone for fluid samples in the upper part of the cell, a cone-shaped hole was made (Figure 3) of 18 mm^2^ surface. The liquids are injected into and removed from the cavities with a micropipette.

#### 2.3.4. Preparation of Self-Assembled Monolayer (SAM) of 11-Mercaptoundecanoic Acid

The same coating protocol was used for both SPR and LW-SAW assays. Briefly, prior to use, the gold surfaces used in the SPR and LW-SAW assays were washed with ultrapure water and dried with nitrogen gas. The cleaned surfaces of the gold chips were incubated with 1 mM solution of 11-MUA in absolute ethanol for 24 h, subsequently rinsed with ethanol and ultrapure water for the removal of the residual 11-MUA molecules and finally dried by passing through an argon stream. The SAM functionalized chips were then mounted onto SPR and LW-SAW cells, respectively.

### 2.4. Antibody Immobilization onto SAM Functionalized Surfaces

The antibody was immobilized via its primary amine groups (lysine residues), using the EDC/NHS chemistry [16]. The purpose was to obtain an increased binding capacity of the antibody-functionalized surface. Accordingly, the experiments were carried out as follows:

- The activation of the terminal carboxylic groups of the linker via EDC/NHS esters was performed by three time injecting 100 μL of a 1:1 mixture of 0.4 M EDC and 0.1 M NHS into the cuvette for 15 min, thus avoiding long time incubations of EDC/NHS mixtures which could have led to the hydrolysis of the unstable NHS esters;
- Anti-AFB1 antibody solutions were prepared in a 10 mM acetate buffer (pH = 4.5); the value of the working pH was chosen after performing several pre-concentration experiments at pH 3.5, 4.5, 5.5 and 6, in order to avoid repulsive interactions between the negatively charged carboxyl groups of the sensor’s functionalized surface and the negatively charged antibody.
- Anti-AFB1 antibody was immobilized by injecting 50 μL of 1:1000 diluted stock solution (1 µg/mL) for 40 min in the SPR and LW-SAW assays;
- The remaining active sites of the sensors were blocked by injection of 50 μL of 1 M ethanolamine (pH = 8.5) for 15 min, followed by rinsing with PBS 0.1 M, pH 7.5.

### 2.5. AFB1-BSA/Anti-AFB1 Antibody Binding Assays

The AFB1-BSA/anti-AFB1 antibody interaction assays were performed according with two protocols:

(1) 50 μL of AFB1-BSA in PBS solutions (pH 7.5), with concentrations ranging from 3 up to 100 nM for the SAW sensor and 6.5 nM up to 265 nM for the SPR sensor (in PBS, pH 7.5) were injected onto sensors surface. The solutions were kept in contact with the immobilized antibody for 40 min during the association phase in SPR assays and for 20 min in the LW-SAW assays, in order to reach the thermodynamic equilibrium. Then, the solutions were drained out and 50 μL PBS was injected for the dissociation phase. Since the SPR protocol uses a double channel system, in order to extract any potential contributions to the SPR signal resulting from the interaction between the protein part of the bioconjugate and the SAM modified surface, the antibody was immobilized in both sample and reference channels. During the interaction, a volume of 50 μL AFB1-BSA solution was injected in the sample cell, while 50 μL of BSA solution of the same concentration was passed through the reference cell. In these conditions, the increase of SPR signal after subtracting the reference signal from the sample signal can be assigned only to the binding of the AFB1 part of the bioconjugate to the antibody epitope.
   In the case of the LW-SAW sensor, separate assays with BSA were performed as blank experiments and the LW-SAW signal corresponding to BSA non-specific binding was extracted from the LW-SAW signal of AFB1-BSA. Each interaction experiment was followed by a dissociation step with 0.1 M PBS pH 7.5 and a regeneration step with 0.1 M HCl incubated for 10 min over the sensor surface.
(2) The same ranges of AFB1-BSA concentrations as depicted above was kept for both SPR and SAW assays but, in order to avoid the time-consuming regeneration steps required after each interaction assay, we used a successive additions format. Thus, the dissociation and the regeneration sequences were carried out after the stabilization of the SPR/LW-SAW signal corresponding to the highest concentration of AFB1-BSA. All SPR/LW-SAW measurements were made at a constant temperature (25 °C).

## 3. Results and Discussion

### 3.1. Paired Interaction Assays with SPR and LW-SAW Sensors

#### 3.1.1. Interaction Monitoring with the SPR Sensor

In our SPR batch system, the hydrodynamic properties were controlled by constant agitation of the bulk solution in contact with the sensor surface. The changes in the resonance signal expressed in millidegrees were followed as a function of time and presented as a sensorgram. The SPR system allowed calculating the surface density of the immobilized antibody. The purpose was to obtain an increased binding capacity of the antibody functionalized surface. This was checked through SPR measurements and quantified by the value of R_max_ (or Δθ_SPR max_), corresponding to the maximum amount of AFB1-BSA which can bind to the immobilized anti-AFB1 antibody. Since the same immobilization protocol was achieved for both LW-SAW and SPR sensors, we assumed that the same amount of surface bound antibody was obtained. We achieved the binding experiments at the optimum binding capacity R_max_ of 200 m°, corresponding to an available surface density of anti-AFB1 antibody of 1.6 ng·mm^−2^.

#### 3.1.2. Optimization of the Regeneration Procedure during the SRP Assays

The regeneration procedure of the anti-AFB1 antibody modified SPR sensor consisted in injecting 50 µL of 0.1 M HCl only, followed by washing with ultrapure water prior the next experiment. The regeneration time was 15–20 min in the case of the first protocol, with regeneration after each binding assay and 30–60 min for the successive addition protocol; since the latter gave around 90%–96% recovery of the initial SPR signal, it was used in the further binding experiments with the SPR sensor. 

#### 3.1.3. Interaction Monitoring with the LW-SAW Sensor

In order to examine the changes in the morphology of gold surface due to antibody immobilization, the Atomic Force Microscopy (AFM) investigations were performed prior studying the Ab–Ag interaction. The AFM images reveal topographic information with nanometric resolution for each point assigned a specific height into a colour scale. The resulting images are formed by combining the topographic information with specific slope shading effects.

**Figure 4:**
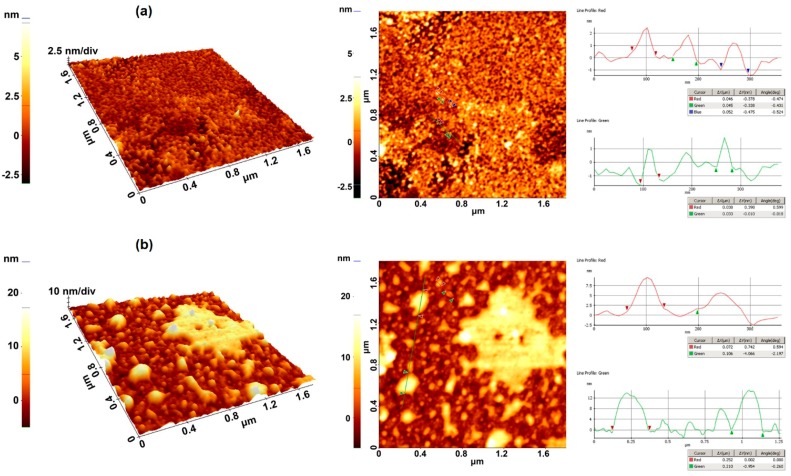
3D (**left**) and 2D (**right**) AFM images for (**a**) bare gold thin films and (**b**) antibody covalently immobilized onto 11-MUA coated gold thin film (1.8 µm × 1.8 µm).

In Figure 4a, the image of the bare gold thin film is shown. A smooth, uniform surface without cracks or droplets can be observed, and the roughness (RMS) is less than 1 nm for the selected area of 1.8 µm × 1.8 µm. Gold nanoparticles with regular shape and sizes of 40–50 nm uniformly cover the surface. Noticeably, the gold film with anti-AFB1 antibody immobilized using the 11-MUA thiol SAM (Figure 4b) exhibits a higher height than bare gold film of approximately 5 nm. This is in agreement with the size of the antibody (*ca.* 4 nm). The covalent immobilization method leads to a relatively uniform distribution of the Ab on the surface of the LW-SAW sensor.

**Figure 5:**
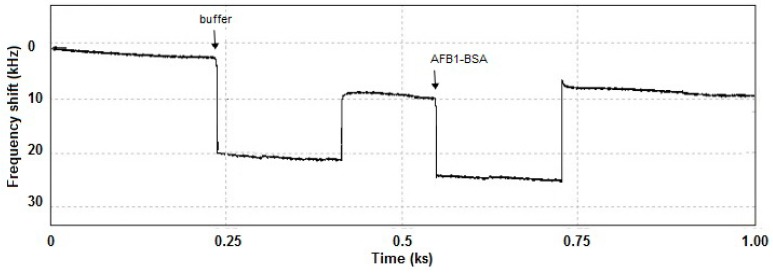
Frequency shifting associated with the amount of the surface-bound AFB1-BSA recorded in PBS solution (pH = 7.5) with the LW-SAW sensor.

The immuno-interactions can be mainly detected as mass variation at the sensor surface because these interactions produce frequency shifts. The LW-SAW cell was designed for performing similar assay as with the SPR cell, both sensors being sensitive mainly to slight mass changes near the functionalized surface. In contrast with the stabilization of the SPR sensors response, LW-SAW sensors usually require a shorter time to reach a stable value. Therefore, the oscillation frequency shift of the acoustic wave was analyzed for different incubation times of AFB1-BSA on the sensor surface (*i.e.*, 3, 10, 20, 30 and 40 min). A significant drop of the frequency has been observed in the first 3 min of AFB1-BSA incubation, while after 20 min the signal reached a stable value, as it can be seen in the LW-SAW sensorgram presented in Figure 5. The measurements time in the LW-SAW assay was reduced at a half of the corresponding measurement time from the SPR assay (40 min.). The time to reach the thermodynamic equilibrium of Ab/Ag is the same on both LW-SAW and SPR sensors, but the SPR sensor requires a longer time to stabilize the signal. It is well known that in SPR detection, a longer stabilization time of the response was observed due to the need of the equilibration of the sensor surface [16]. To ensure the accuracy of the SPR control experiments, we decided to perform the measurements assigned to the association step for 40 min.

The magnitude of the frequency shift assigned to the specific AFB1-BSA/anti-AFB1 antibody binding was proportional to the concentration of the injected AFB1-BSA, demonstrating the selectivity of the antibody functionalized surface.

#### 3.1.4. Optimization of the Gold Layer Thickness of the LW-SAW Sensor

A thin gold layer was deposited onto the PMMA waveguide layer, between the IDTs, within an area of 9 × 10 mm. The gold layer was used as a substrate for the formation of the thiol-based SAM prior to immobilization step of the Ab. We have tested gold layers with thickness varying between 10 and 50 nm and the immobilization of anti-AFB1 antibody was achieved on all LW-SAW sensors.

**Figure 6:**
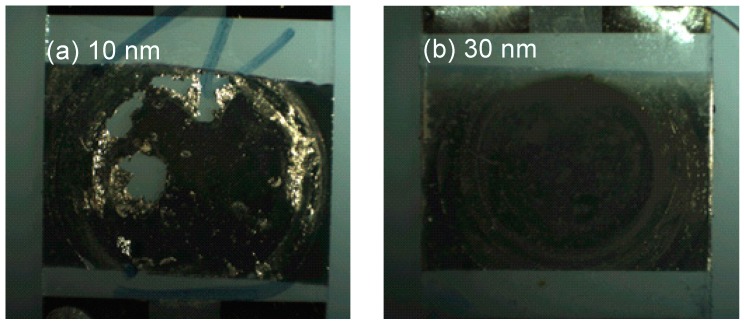
Microscopic images of the gold layers (**a**) 10 nm; (**b**) 30 nm.

We have noticed that some areas of the gold layer were exfoliated during the immobilization process when a 10 nm thin layer was used (Figure 6a). While increasing of the gold layer thickness may yield a more stable surface during the immobilization, interaction and regeneration steps, a thicker layer could cause significant perturbations of the LW-SAW oscillation frequency. Thus, a 50 nm thickness leads to a decrease with 3 MHz of the oscillation frequency, which was considered too large for the oscillation frequency of this sensor (~69 MHz). For this reason, we could not use the same gold layer thickness as for the commercial SPR gold chip (50 nm). Therefore, a 30 nm thickness was considered a good compromise between a stable gold surface and a consistent response of the SAW sensor, and was used for all subsequent experiments.

#### 3.1.5. Optimization of the Regeneration Procedure during the LW-SAW Assays

First calibration tests were performed by successive incubation steps with AFB1-BSA, followed by the regeneration of the LW-SAW immunosensor. The regeneration procedure of the LW-SAW immunosensor consisted in the incubation of 100 µL of 0.1 M HCl on the sensor surface for 10 min, followed by washing with PBS solution prior the next experiment. A 38.7% decrease of the reference signal was observed after a first step of regeneration, while after the second and the third regeneration step the decrease of the reference signal was around 40%–47%. Taking into account all these aspects, the calibration procedure was modified by using the successive additions method, in the concentration range at 3–100 nM AFB1-BSA, with one final regeneration step after signal saturation. The regeneration procedure has been modified as follows: 50 µL of 0.1 M HCl were added on the surface of the sensor and incubated for 10 min, then the HCl solution was removed and the sensor surface was washed with ultrapure water.

### 3.2. Calibration of the SPR and LW-SAW Sensors

It is generally accepted that shape of the SPR response for the antibody–antigen interactions displays a hyperbolic dependence on the concentration of the injected target (here AFB1-BSA), being mainly influenced by the affinity of the target for the immobilized receptor. However, the position of saturation zone of the hyperbolic curve depends also on the sensor’s sensitivity to the changes occurring near the functionalized surface and might differ from SPR to SAW sensors, for example. We noticed that Langmuir isotherm-like functions could be fitted to both SPR and LW-SAW experimental data. Even though there are different thicknesses of the gold layer for the LW-SAW and SPR sensors, we considered it to be suitable to compare the performances of the binding assays obtained with the two sensors, since the immunoassay protocols are similar. We expected an increased sensitivity of the LW-SAW sensor towards the SPR sensor because the detection principle of the LW-SAW sensor is based on changes in mass, density and viscosity occurring near the surface, while the SPR detection principle lies in changes in the refractive index due mainly to mass variation near the surface. Therefore, we have chosen to compare the performance of SPR and LW-SAW sensors in terms of sensitivity, limit of detection (LOD) and dynamic range. To do this, we calculated the normalized sensor response (R_norm_), defined as the ratio R_eq_/R_max,_ where R_eq_ represent the sensor’s response after reaching a constant value and R_max_ being the sensor signal corresponding to the maximum concentration of the injected AFB1-BSA which can bind the immobilized antibody.

In the case of SPR sensors, the Langmuir binding isotherm can be fitted only on interactions with 1:1 stoichiometric ratios. One can discriminate among mono- and multi-valent immobilized receptors when the interaction profile target/receptor does not fit the Langmuir pattern or when the normalized response at equilibrium R_eq_/R_max_ exceeds unity [10]. For a bimolecular interaction with molecules A (target) and B (immobilized receptor) forming the complex AB, the equilibrium association constant (or affinity constant) K_a_ and dissociation constant K_d_ are given by Equations (1) and (2):

(1) $$K_{a}=\frac{{\left[AB\right]}_{eq}}{{\left[A\right]}_{eq}{\left[B\right]}_{eq}}({\text{M}}^{\text{−1}})$$

(2) $$K_{d}=\frac{1}{K_{a}}(\text{M})$$

The SPR responses, R and R_max_ are directly correlated with the molecular weight of the bound target and with the concentrations of the superficial complexes [AB] and [AB]_max_; since the surface concentrations [AB]_max_ and [B]_0_ are equal only for 1:1 interactions (with all the binding sites being occupied), one obtains:

(3) $$\frac{R}{R_{\max}}=\frac{\left[AB\right]}{{\left[B\right]}_{0}}$$

where the R/R_max_ ratio represents the normalized response R_norm_

K_a_ as a function of SPR response can be obtained by combining Equations (1) and (3) in equilibrium conditions:

(4) $$K_{a}=\frac{R_{eq}}{{\left[A\right]}_{eq}\left(R_{\max}-R_{eq}\right)}$$

Equation (4) could be rearranged in order to correlate R_eq_ with the equilibrium concentration of target, [A_eq_].

(5) $$R_{eq}=\frac{K_{a}{\left[A\right]}_{eq}}{1+K_{a}{\left[A\right]}_{eq}}\cdot R_{\max}$$

or

(6) $$\frac{R_{eq}}{R_{\max}}=\frac{K_{a}{\left[A\right]}_{eq}}{1+K_{a}{\left[A\right]}_{eq}}$$

**Figure 7:**
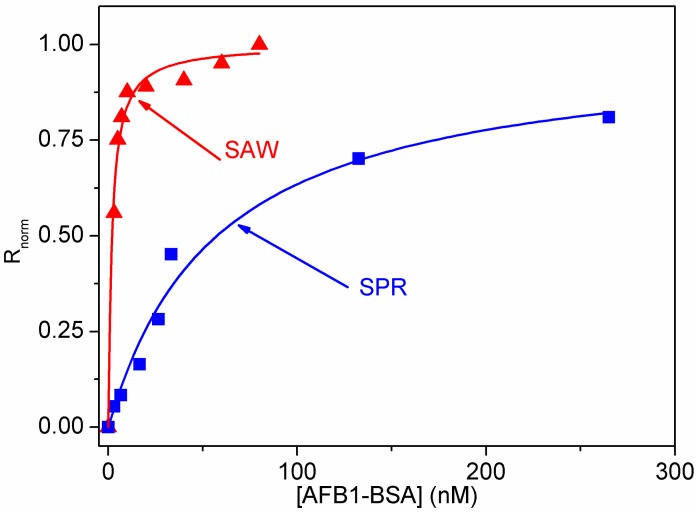
Langmuir-like binding curves obtained with the SPR and LW-SAW sensors pH = 7.5 and T = 25 °C.

Equation (6) provides the analytical form of R_eq_ or R_norm_ dependence on [A]_eq_ according to Langmuir pattern [10]. Since the amount of the surface-bound target is negligible with respect to the target amount remaining in the solution, one can approximate [A_eq_] = [A]_0_, where [A]_0_ is the concentration of the injected target. Given the shorter time necessary for reaching an equilibrium value with the LW-SAW sensor compared to the SPR sensor, one can assume that the K_a_ parameter has the significance of an affinity constant for the binding curved obtained within the SPR assays, while the Langmuir-like curve obtained within the LW-SAW measurements was used to build the calibration plot (Figure 7).

The estimated affinity constant derived from the Langmuir curve was K_a_ = (1.700 ± 0.016) × 10^7^·M^−1^ (with *r* = 0.9905 and a significance level α = 0.05), a value that matches well with the reported affinity constants for antibody/antigen binding [17].

The detection of the AFB1-BSA target can be carried out by extracting the linear part of the Langmuir-like binding curve. The straight line equations fitted on the experimental data R_norm_ *vs.* AFB1-BSA concentration were: Y = (0.0286 ± 0.0023) + (0.1533 ± 0.0147)X with *r* = 0.9705, α = 0.05 for the LW-SAW sensor and Y = (0.0111 ± 0.0087) + (0.010033 ± 0.00061)X with *r* = 0.9945, α = 0.05 for the SPR sensor. The linear fit for the range 3–10 nM displayed the highest sensitivity: 0.1533 normalized response units (n.r.u.)/nM in the range 3–10 nM for the LW-SAW sensor while the SPR sensor displayed the highest sensitivity in the range 3–26.5 nM: 0.01 n.r.u/nM. The two sensors provide nanomolar dynamic ranges as observed from the Langmuir-like binding curves. The limit of detection expressed as the concentration of AFB1-BSA for an analytical signal of 10% from the saturation signal of the sensor was 10 nM for the SPR sensor and 0.6 nM for the LW-SAW sensor. However, it can be noticed that the dynamic range of the SAW sensor is significantly narrower than the one obtained with the SPR sensor, probably because the SAW sensor’s response reached a saturation value at a concentration below the maximum binding capacity of the immobilized antibody.

Remarkably, our developed SAW sensor exhibited a lower LOD than the one obtained with the conventional SPR sensor, as well as an increased sensitivity towards the SPR detection for the same immunoassay format. These aspects, corroborated with the significant reduction of the analysis time recommend the implementation of our LW-SAW assay in the detection and monitoring of other relevant high molecular weight targets such as DNA and proteins.

## 4. Conclusions

In this work, we developed a LW-SAW sensor together with a label-free immuno-interaction protocol for the detection of high molecular weight targets in liquid samples. This method was first checked with a SPR sensor and could be amenable for the detection of low molecular targets in surface competition assays, which are currently in use for ELISA-based analysis. The obtained affinity constant for the AFB1-BSA/anti-AFB1 antibody with the SPR sensor matched well with the reported values for common antibody–antigen interactions. This fact suggests that the conjugation of AFB1 to a carrier protein has not a significant influence on the affinity of AFB1 for the corresponding antibody. Thus, the immunoassay formats involving the high molecular weight bioconjugate AFB1-BSA can be further used for developing biosensors for AFB1 bound to plasma proteins. The level of AFB1-protein adducts in blood being directly correlated to the level AFB1-DNA adducts, one can use our immunoassay format for quantifying biomarkers of cumulative AFB1 exposure and to detect AFB1 metabolites from contaminated meat in food products. We are aware that the sensitivity and the dynamic range of our sensors can appear not competitive with other label-free technologies. Despite that, the designed SAW cell with the implemented binding protocol could be easily adapted to portable low-cost platforms, finally making them suitable for implementation in point-of-care analysis.
